# Effects of Buffer Concentration on the Sensitivity of Silicon Nanobelt Field-Effect Transistor Sensors

**DOI:** 10.3390/s21144904

**Published:** 2021-07-19

**Authors:** Chi-Chang Wu, Min-Rong Wang

**Affiliations:** 1 Department of Electronic Engineering, National Chin-Yi University of Technology, Taichung 411030, Taiwan; 2 Department of Electronic Engineering, Feng Chia University, Taichung 407802, Taiwan; m0821705@o365.fcu.edu.tw

**Keywords:** silicon nanobelt, FET sensor, pH sensing, alpha fetoprotein, ionic strength, Debye screening length

## Abstract

In this work, a single-crystalline silicon nanobelt field-effect transistor (SiNB FET) device was developed and applied to pH and biomolecule sensing. The nanobelt was formed using a local oxidation of silicon technique, which is a self-aligned, self-shrinking process that reduces the cost of production. We demonstrated the effect of buffer concentration on the sensitivity and stability of the SiNB FET sensor by varying the buffer concentrations to detect solution pH and alpha fetoprotein (AFP). The SiNB FET sensor was used to detect a solution pH ranging from 6.4 to 7.4; the response current decreased stepwise as the pH value increased. The stability of the sensor was examined through cyclical detection under solutions with different pH; the results were stable and reliable. A buffer solution of varying concentrations was employed to inspect the sensing capability of the SiNB FET sensor device, with the results indicating that the sensitivity of the sensor was negatively dependent on the buffer concentration. For biomolecule sensing, AFP was sensed to test the sensitivity of the SiNB FET sensor. The effectiveness of surface functionalization affected the AFP sensing result, and the current shift was strongly dependent on the buffer concentration. The obtained results demonstrated that buffer concentration plays a crucial role in terms of the sensitivity and stability of the SiNB FET device in chemical and biomolecular sensing.

## 1. Introduction

Chemical and biological sensors have attracted much attention because of their wide applicability in daily life [1,2,3,4]. The demand for reliable, ultrasensitive, and portable sensors is increasing in fields such as disease diagnostics, human health, and environmental monitoring [5,6]. For example, the sensor-detection of trace amounts of cancer markers benefits patients in receiving preventative health care and early-stage treatment that can greatly increase cancer survival rates. Conventional methods of sensing biomolecules use enzyme-linked immunosorbent assays and polymerase chain reactions [7,8,9,10], which sense antigen or antibody levels and DNA fragments, respectively. Both methods require fluorescent molecule labeling on the sensing targets. Other disadvantages include the requirement of complicated pretreatment before sensing, unportable devices, and relatively insensitive sensing.

pH values strongly affect human health, and fluctuations or variations of pH values in the body can cause various diseases; therefore, pH monitoring is paramount for understanding human physiology and pathology [11]. In addition, pH plays a crucial role in chemical and biological reactions [12,13]. For example, enzymes catalyze biomolecules at optimal pH ranges, and enzyme activity is reduced if the pH value exceeds a certain range. An extreme pH value results in enzyme denaturation [14]. Therefore, a sensitive, rapid-response, cost-effective, and portable sensor system is urgently required for pH monitoring in various applications [15]. Conventional techniques used for pH sensing employ electrochemical methods, including potentiometric, conductimetric, capacitive, and resistive sensors [16,17]. The potentiometric method is most commonly used for pH sensing and comprises a sensitive electrode and reference electrode. A redox reaction occurs at the metal oxide on the sensitive electrode surface in a solution; thus, detection is conducted through the measurement of the potential difference between the reference and sensitive electrodes in a solution of an unknown pH [18]. The main disadvantages of the potentiometric pH sensors are the difficulty of miniaturizing the reference electrode and the instability of the potential during long-term operation.

Field-effect transistor (FET)-based sensors have drawn increasing attention on account of their favorable properties [19,20,21,22]. Compared with the aforementioned conventional methods, FET-based sensors are advantageous for their small dimensions, low cost, fast response, label-free operation, and integration capability [23,24]. An FET-based sensor detects chemical reactions or biomolecular bonds through measurement of the current or voltage shift caused by the generation of excess charges, which exerts an electrical field into the FET channel [25]. FET-based sensors can be applied in fields such as chemistry, food processing, pharmaceuticals, environmental science, and biomedical engineering. The fabrication of FET-based sensors is fully compatible with the manufacturing of modern commercial metal–oxide–semiconductor (CMOS) FETs, and thus, they can be cost-effectively mass produced [26].

Due to these advantages, several FET-based sensors have been adapted to chemical and biological detection. Moreover, many types of semiconductor materials with quasi-one-dimensional nanostructures, such as nanobelts and nanowires [27,28,29,30], have been applied to FET-based sensors. FET-based sensors in conjunction with nanostructures have demonstrated exceptional sensitivity as a result of their large surface-area-to-volume ratios. Thus, the ultrasensitive, specific, and fast-response sensing of chemical and biological targets has become feasible [31,32].

The dependence of buffer concentration and nanowire sensitivity is still controversial. Some research groups claimed that the mechanism of chemical and biological sensing using a FET-based sensor relies on the protonation or deprotonation phenomenon of the functional groups at the solution–dielectric interface [33]. Therefore, the ionic concentration in the buffer solution plays a vital role in the effectiveness of chemical and biological sensing. High ionic concentrations reduce the sensitivity of FET-based sensors and in turn, their applicability [34]. On the other hand, S. Zafar reported that the sensing mechanism of the nanowire in ionic solution was complicated, and their sensing results showed that the pH sensitivity was independent of the buffer concentration [35].

Therefore in this study, a single-crystalline (SC) silicon nanobelt field-effect transistor (SiNB FET) device was fabricated as a chemical and biological sensor. The nanobelt was formed using a local oxidation of silicon technique, a self-aligned process in which the nanobelt can be reduced to a smaller size during formation without the use of expensive advanced lithography. The fabrication of the SiNB FET device is fully compatible with the industrial manufacture of CMOSs, allowing for cost-effective mass production. To verify the effect of buffer concentration on the sensitivity of the SiNB FET, a buffer solution of varying concentrations was employed to inspect the sensing capability of the sensor. Our results might help to clarify the role of ions in the buffer solution when chemical or biological sensing. In addition, the stability and repeatability of the sensor in different solutions were also examined. For biomolecule sensing, alpha fetoprotein (AFP) was employed as the detection target, and the sensitivity and stability of the sensor were also tested in various buffer concentrations in detecting AFP target.

## 2. Materials and Methods

The SC SiNB FET sensor devices were fabricated in the Taiwan Semiconductor Research Institute (Hsinchu). Analytical-grade ethanol (C_2_H_5_OH, 99.5%), (3-Aminopropyl)triethoxysilane [H_2_N(CH_2_)_3_Si(OC_2_H_5_)_3_; APTES; 22.137 g/mol], and phosphate-buffered saline (PBS; 120 mM NaCl, 2.7 mM KCl, 10 mM phosphate buffer) were purchased from Sigma-Aldrich (St. Louis, MO, USA). Glutaraldehyde (OHC(CH_2_)_3_CHO; GA; 25%) was purchased from MP Biomedicals (Santa Ana, CA, USA), and sodium phosphate monobasic monohydrate (NaH_2_PO_4_·H_2_O) and sodium phosphate dibasic (Na_2_HPO_4_) were purchased from J. T. Baker (Phillipsburg, NJ, USA). The antibody and antigen for AFP were purchased from Blossom Biotechnologies (Taipei, Taiwan).

### 2.1. SiNB FET Device Fabrication

**Figure 1:**
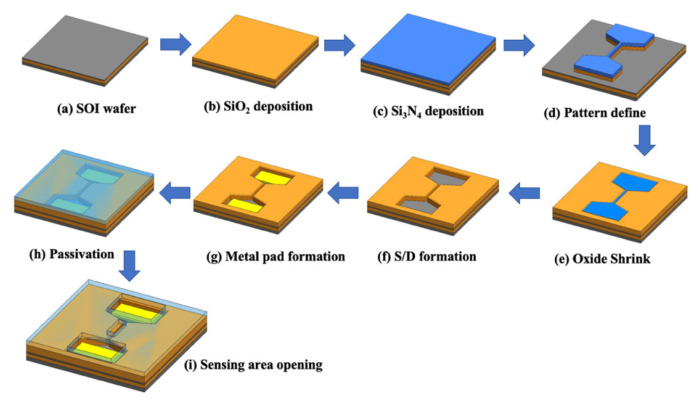
Schematic of the SC SiNB FET fabrication process.

A schematic of the process of fabricating the SC SiNB FET sensor is illustrated in Figure 1. The SC SiNB FET devices were fabricated using commercially available 6-inch silicon-on-insulator (SOI) wafers as the substrate (Figure 1a). The SOI wafer had a 50-nm-thick SC silicon film and 150-nm-thick buried oxide film. First, a stacked film of SiO_2_ and Si_3_N_4_ layers was deposited on the SOI wafer (Figure 1b,c). Then, the SiO_2_/Si_3_N_4_ stack layers and the SC silicon film beneath the stack layer were patterned and dry etched to define the active region (Figure 1d). An oxidation process was subsequently conducted to oxidize the SC silicon. The SC silicon exposed to air was then oxidized into SiO_2_ film. The SC silicon film was capped underneath the Si_3_N_4_ layer to prevent oxidization because the Si_3_N_4_ capping layer prevents oxygen diffusion into the silicon film. In addition, lateral oxidation of the silicon was observed at the edge of the Si_3_N_4_ capped SC silicon film, causing the width of the SC silicon nanobelt to shrink (Figure 1e). As a result, the formed linewidth of the SC silicon nanobelt was two-thirds smaller than the critical linewidth capable for the lithography system. This lateral oxidation process has the capacity to easily and stably form a small silicon nanobelt without the use of expensive and advanced exposure tools.

After the nanobelt shrinking process, the SiO_2_/Si_3_N_4_ stack layers were removed. As^+^ ion implantation at a dosage of 10^16^ and energy of 10 KeV and rapid thermal annealing were conducted to form the source/drain region (Figure 1f). The stacked Al–Si–Cu/TiN metal films were deposited using the sputter system, followed by contact pad defining and metal etching (Figure 1g). To protect the sensor device, TEOS SiO_2_ and Si_3_N_4_ films were deposited sequentially (Figure 1h), and the sensing area was etched back to expose the nanobelt for sensing (Figure 1i). Finally, the fabricated SC SiNB FET devices were spin coated with a photoresist (PR) layer and stored in an N_2_ ambient environment to prevent nanobelt oxidation and contamination.

### 2.2. Surface Modification of the SC SiNB FET Device

**Figure 2:**
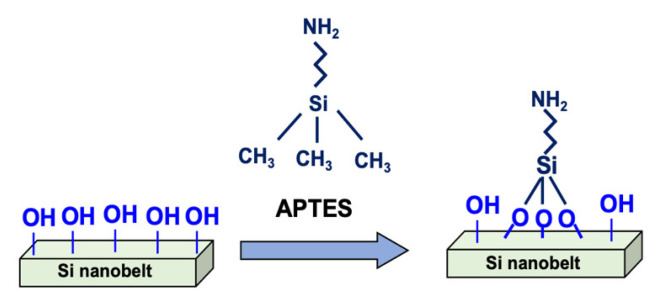
Schematic representation of surface modification on the nanobelt surface.

Prior to surface modification, the PR layer was removed by immersing the device in acetone for 10 min. The device then underwent ultrasonic cleaning for 10 min with ethanol, followed by a rinse process using high-purity deionized (DI) water. PR removal is essential for sensing because the PR residue reduces the efficiency of surface modification, leading to device instability. In addition, the PR removal process also cleans the sensor surface, preventing contamination. Next, an oxygen plasma treatment was conducted for 15 min to modify the sensing area and ensure an OH^−^ rich surface. The sensor devices were then immediately immersed in an APTES and ethanol mixed solution (2:98) for 30 min at 37 °C. The samples were rinsed with DI water and heated at 120 °C for 30 min. This process induces an APTES reaction with the surface silanol groups, and hence, silanol and amino groups are both modified on the nanobelt surface. The silanol and amino groups on the surface are vital in pH sensing because of their proton donor and acceptor roles. Figure 2 illustrates the process of surface modification on the nanobelt surface.

### 2.3. Preparation of Buffer Solutions of Varying pH

The buffer solution used in this study was formed from NaH_2_PO_4_·H_2_O and Na_2_HPO_4_. Each powder was dissolved into DI water to form a 1-mM solution. A pH meter (6173 pH; Jenco Electronics, Grand Prairie, TX, USA) with ±0.01 precision was used to measure the pH of the buffer solution. Initially, the pH meter was calibrated using standard solutions (pH 7.00, 4.01, and 10.01; Jenco Electronics, Grand Prairie, TX, USA). In the next step, a NaH_2_PO_4_·H_2_O solution of pH 4.3 was used as the base solution, and a Na_2_HPO_4_ solution of pH 9.0 was titrated to adjust the buffer solution to various pH values ranging from 6.4 to 7.4.

### 2.4. Surface Modification and Biografting for AFP Sensing

In this study, AFP was employed as the target for biosensing. To sense AFP using the SC SiNB FET, a surface modification process was undertaken. After oxygen plasma cleaning and the APTES process described in Section 2.2, the sensor was immersed in 2.5% GA solution for 30 min at room temperature, followed by rinsing with DI water and nitrogen drying. This functionalization process was used to link the GA in the amino groups, thus exposing aldehyde groups on the SiNB FET surface for AFP biografting. The antibody for AFP (anti-AFP) was diluted to 500 ng/mL with PBS and then placed on the SiNB FET surface for 10 min to ensure efficient binding, followed by a rinse with PBS to remove excess anti-AFP. The device was immersed in bovine serum albumin solution to block unreacted terminals. The AFP antigen of 10 ng/mL was then injected into the microfluidic channel and run through the sensor region to bind with anti-AFP; this was followed by a rinse with PBS solution to remove unreacted AFP. The microfluidic channel was made of polydimethylsiloxane (PDMS) and its fabrication has been described in a previous paper [36]. The real-time electrical response of the SC SiNB FET sensor was measured simultaneously using an Agilent 4156C instrument (Agilent Technologies, Santa Clara, CA, USA).

### 2.5. Measurement and Analysis of the SC SiNB FET Devices

The electrical measurement of the SiNB FET sensor devices was conducted using the Agilent semiconductor parameter analyzer. The drain current (*I_D_*) versus gate voltage (*V_G_*) and *I_D_* versus drain voltage (*V_D_*) were characterized to evaluate the performance of the devices. To measure the real-time electrical response of the SiNB FET sensor, constant *V_D_* and *V_G_* were applied to the device during measurement, and the synchronous *I_D_* was recorded every 5 s to avoid thermal drifting of the FET device. The recorded current could observe the response of the SC SiNB FET sensor in the buffer solution. Cross-sectional images of the silicon nanobelt were captured through transmission electron microscopy (TEM; JEM-2010F; JEOL, Tokyo, Japan).

## 3. Results and Discussion

### 3.1. Basic Characteristics of the SC SiNB FET Device

**Figure 3:**
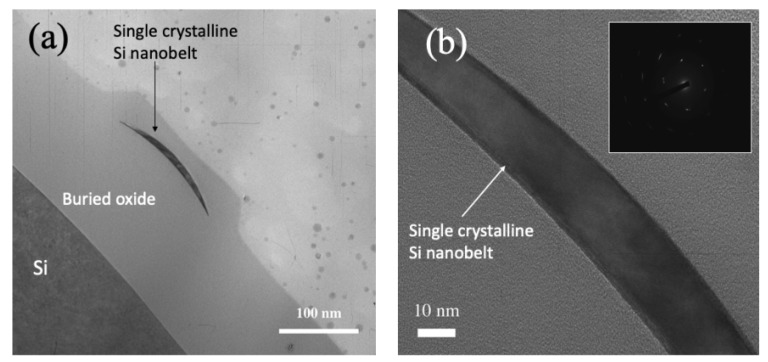
TEM images of the SC silicon nanobelt. (**a**) Cross-sectional image of the SC nanobelt using the local oxidation technique. (**b**) Enlarged image of the SC nanobelt. Inset is the deflection pattern of the silicon nanobelt.

The cross-sectional TEM images of the SC silicone nanobelt are depicted in Figure 3. The nanobelt exhibited a bending shape, which was caused by the invasion of the SiO_2_ film into the side wall of the SC silicon during lateral oxidation. This phenomenon is known as the bird’s beak effect of lateral oxidation [36]. As a result, the residual SC silicon film was bent and shrunk to a width smaller than the critical size for the lithography technique. In this case, a 350-nm lithography technique was employed, and finally, the size of the SC nanobelt was shrunk to 150 nm wide and 30 nm thick using lateral oxidation. The electron diffraction pattern illustrated in the inset of Figure 3b provided crystallographic information, validating the formation of SC.

**Figure 4:**
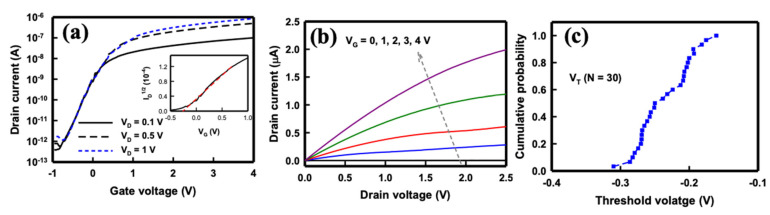
Electrical properties of the SiNB FET device. (**a**) Drain current versus *V_G_* at *V_D_* = 0.1, 0.5, and 1 V. (**b**) Drain current versus *V_D_* at *V_G_* = 0, 1, 2, 3, and 4 V. (**c**) Cumulative probability of threshold voltage (n = 30).

The NB sensor behaved as an electrical field-effect device in which the electron carriers laterally traveling through the 1.6-μm-long nanobelt were effectively controlled by the longitudinal electrical field from the gate voltage. Therefore, the performance of the SC SiNB FET sensor device was assessed through the application of different drain voltages (*V_D_*) and gate voltages (*V_G_*), and the drain current (*I_D_*) was measured accordingly. In this study, *V_G_* was applied using backside gate potentials. Figure 4 depicts the basic electrical characteristics of the SC SiNB FET sensor devices. The behaviors of the SiNB FET device were consistent with that of the n-channel MOS FET devices. The *I_D_* with respect to the *V_G_* at different *V_D_* is presented in Figure 4a. The on−off current ratio of this SC SiNB FET was determined using the ratio of the highest and lowest *I_D_* in Figure 4a and could achieve up to six orders of magnitude if the device was operated at *V_D_* = 1 V. The threshold voltage (*V_T_*) could be determined when the SC SiNB FET was operated in the saturation (*SAT*) regime. The drain current of the SC SiNB FET in the *SAT* region can be calculated as follows [37]:

(1) $$I_{D,SAT}=\frac{mW\mu_{eff}C_{ox}}{L}{\left(V_{G}-V_{T}\right)}^{2}$$

where *m* is a function of the doping density in the channel and is generally 0.5 for low-doping densities, *μ_eff_* is the effective mobility of the carriers, *C_ox_* is the oxide capacitance per unit area, *W* is the channel width, and *L* is the channel length. From Equation (1), a plot of the root of *I_D_* (*I_D_*^1/2^) versus *V_G_* can be drawn, and *V_T_* is extracted by extrapolating the curve to 0 *I_D_* [38]. Thus, the *V_T_* of the SiNB FET device was approximately −0.25 V, as illustrated in the inset of Figure 4a. The subthreshold swing (*S.S.*), which indicates the controllability of the gate of the device, is defined as the *V_G_* that must be applied to increase the *I_D_* by 10-fold. The *S.S.* of the SiNB FET device can be derived from Figure 4a and is defined as

(2) $$S.S.={\left(\frac{\partial logI_{D}}{\partial V_{G}}\right)}^{-1}$$

The *S.S.* of the SC SiNB FET device could be extracted from the subthreshold region of the *I_D_*–*V_G_* curve and was approximately 286 mV/decade. This value was higher than that of commercial FET chips because the backside gate was employed in the SC SiNB FET device; thus, the buried 150-nm-thick oxide film served as the gate oxide of the device.

Figure 4b presents the *I_D_*, with respect to the *V_D_* at different *V_G_* ranging from 0 to 4 V, of the SC SiNB FET device. The *I_D_* increased slightly with *V_D_*, indicating that the applied *V_D_* was not the dominant factor for controlling the drain current; instead, the changing *V_G_* considerably altered the current. Therefore, the selection of a suitable applied gate voltage is essential to operate the SC SiNB FET device under the optimal conditions for subsequent pH sensing. Notably, the *I_D_* at the SAT region performed differently from normal FET devices, possibly because of the series resistance caused by its nanobelt structure. Figure 4c illustrates the cumulative probability of *V_T_* of the SiNB FET devices (n = 30). The *V_T_* of the devices was estimated to be −0.23 ± 0.04 V, indicating the stability and reproducibility of the SiNB FET devices formed using lateral oxidation of silicon technology.

### 3.2. pH Sensing of the SC SiNB FET Device

**Figure 5:**
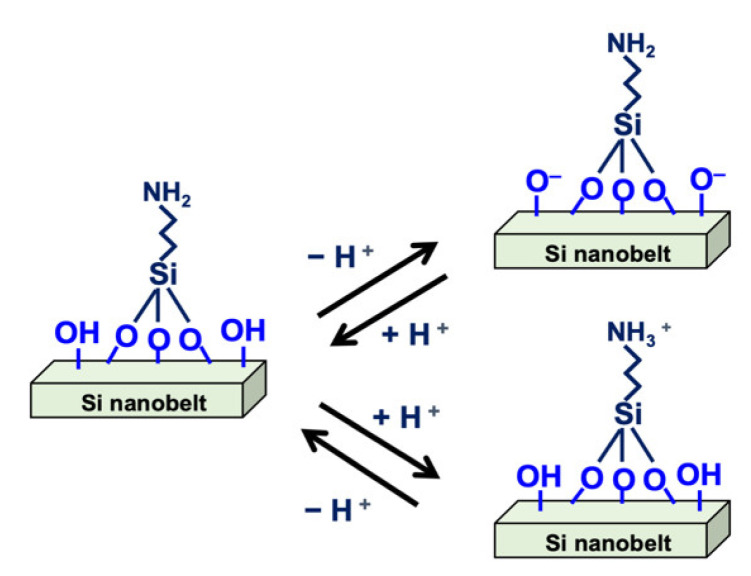
Schematic of protonation and deprotonation of the silicon nanobelt surface.

The prepared buffer solution with various pH values was used to examine the pH-sensing capability of the SC SiNB FET sensor. The sensor surface was initially functionalized with APTES to generate terminal silanol (SiOH) and amino (NH_2_) groups on the nanobelt surface. The functionalization of APTES ensured that the ions were recognized in the solution. These terminated silanol and amine groups are sensitive to the changes in pH values, which enhanced the sensitivity of the pH sensing. Figure 5 illustrates the protonation and deprotonation phenomenon of the nanobelt surface when different dissociation constants (pKa) of the buffer solution were sensed. When a buffer solution with low pKa was added to the surface, either the terminal NH_2_ groups were transformed to NH_3_^+^ or the SiO^−^ groups were transformed to SiOH; conversely, the NH_3_^+^ was transformed to NH_2_ or the SiOH was transformed to SiO^−^ when a high-pKa buffer solution was added to the sensor. The net charge on the nanobelt surface, which was caused by the first-order chemical kinetics of the bond dissociation of the NH_2_ and SiOH terminal groups, induced an additional electrical field resulting in the accumulation or depletion of the carriers in the nanobelt channel, thus affecting the *I_D_* of the SC SiNB FET devices.

**Figure 6:**
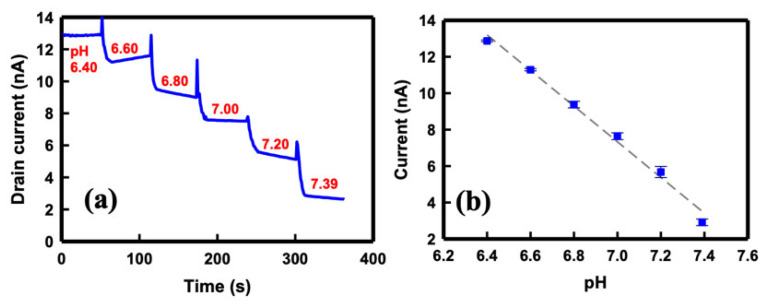
(**a**) Drain current of the SiNB FET sensor under varying solution pH values ranging from 6.40 to 7.39. (**b**) Calibration curve of the drain current as a function of solution pH.

Figure 6a presents the *I_D_* of the SC SiNB FET device when infused with a buffer solution of different pH values on the sensor surface. The pH values of the buffer solution were increased from pH 6.40 to 7.39 with 0.2 in step, and the solution was maintained at a 1X concentration. A buffer solution of pH 6.4 was infused into the detection region by using a microfluidic channel and, after an approximate 50 s wait until the current achieved equilibrium, the solution was expelled and a new solution of pH 6.6 was injected immediately. The other buffer solutions with pH ranging from 6.8 to 7.39 were also infused sequentially using the same procedure. Consistent with the n-channel MOS FET behavior, the *I_D_* current decreased stepwise as the pH of the buffer solution increased. Figure 6b depicts the current exhibiting linear dependence on the pH value of the buffer solution. The devices responded linearly to the pH changes, and the pH sensitivity of the SiNB FET sensor, extracted from Figure 6b, was approximately 10 nA/pH.

**Figure 7:**
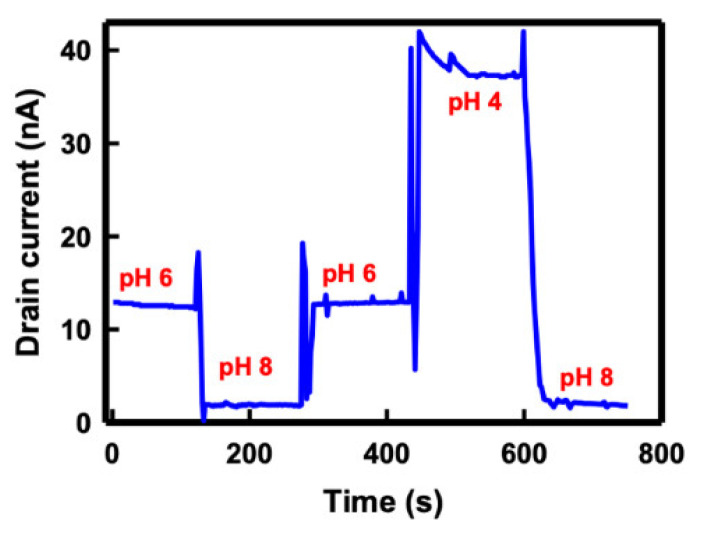
Drain current of the SC SiNB FET sensor under different buffer solution pH values.

In addition to linear sensitivity, the stability of pH sensing of the SC SiNB FET sensor was evaluated by repeatedly infusing the buffer solution to the sensor in a set cycle of pH 6→8→6→4→8. Figure 7 describes the real-time response of the SiNB FET sensor in different buffer solutions. The drain current remained at 13 nA when a pH 6 solution was injected, and it decreased to 1.8 nA in response to a pH of 8. The current returned to approximately 13 nA in a pH 6 buffer solution and then increased sharply when this was replaced with a pH 4 solution. Finally, the current returned to the same level when pH 8 was infused to the sensor. The sensor maintained a stable current level at the same pH value despite sensing the carrying pH of the buffer solutions. This result demonstrated that the SC SiNB FET sensor is reliable for pH sensing.

### 3.3. Effect of the Buffer Ion Concentration on the SC SiNB FET

The ion concentration of the buffer solution plays a crucial role in the sensitivity of the SC SiNB FET biosensor. In an ionic solution, the species with positive or negative charges induces an electrical double layer, and thus, the effective charge to the biosensor is reduced [39]. A parameter describing the effective distance of the ionic solution to influence the nanobelt carrier concentration, known as the Debye screening length (*λ_D_*), can be simplified as follows [40]:

(3) $$\lambda_{D}=0.32\times {\left(I\right)}^{-1/2}$$

where *I* is the ionic strength of the solution and is calculated as

(4) $$I=\frac{1}{2}\overset{n}{\underset{i=1}{\sum }}c_{i}z_{i}{}^{2}$$

where *c_i_* and *z_i_* are the concentration and charge of the ion species, respectively. The equations indicate that the Debye length decreases with an increased ion concentration.

**Figure 8:**
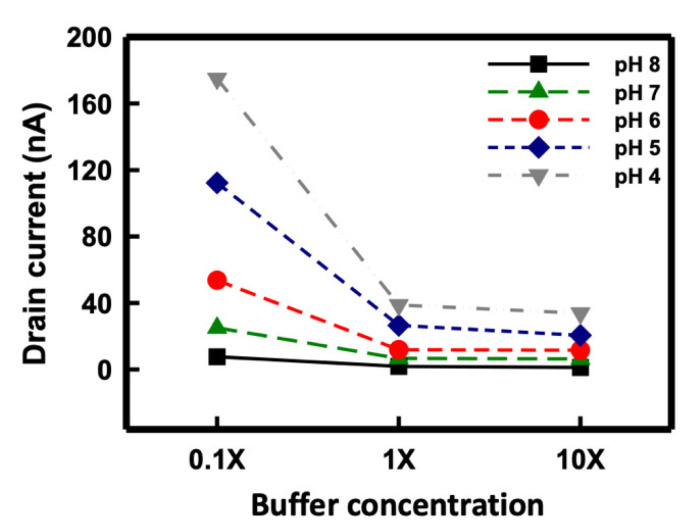
pH sensing under different buffer concentrations using the SC SiNB FET sensor.

Figure 8 presents the drain current of the solution pH under different buffer solution concentrations. The buffer solution was modulated to concentrations of 0.1X, 1X, and 10X, and the sensing response was recorded from pH 4 to 8 by using the SiNB FET sensor. The Debye lengths of the buffer concentrations of 0.1X, 1X, and 10X were estimated to be 2.3, 0.7, and 0.2 nm, respectively. As illustrated in the figure, the sensing response was strongly dependent on the ionic concentration of the buffer solution. The sensitivity of the SC SiNB FET sensor at concentrations of 0.1X, 1X, and 10X was estimated to be 42.2, 10.8, and 7.9 nA/pH, respectively. Table 1 lists pH sensitivity comparison of the relevant reported results using nanowire based FET sensors [41,42,43,44,45]. Sensitivity for pH sensing was ranging from 42 mV/pH to 56 mV/pH because of the Nernst limit. Some works reported that the sensitivity could be improved to exceed the Nernst limit by special structures such as dual-gate operation. As real-time measurement is used for pH sensing in this study, thus the best sensitivity is 42.2 nA/pH, which is around 57.2 mV/pH.

### 3.4. Real-Time Detection of AFP at Various Buffer Concentrations

**Figure 9:**
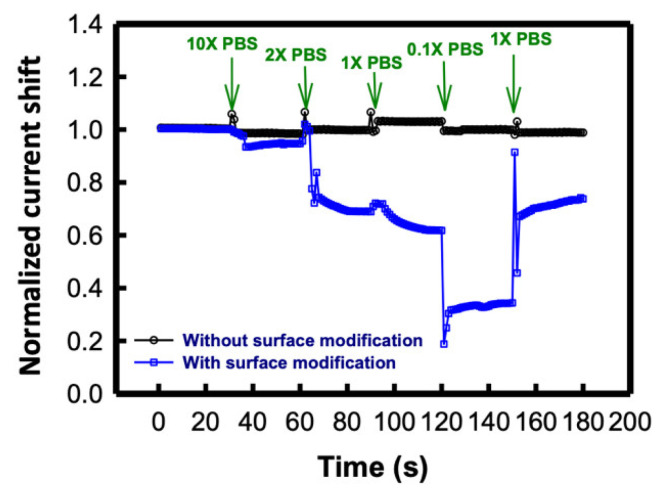
AFP real-time sensing results at different buffer concentrations.

To examine the effect of ionic concentration on the SC SiNB FET device for biomolecule sensing, AFP was employed as the sensing target. AFP is a valuable indicator of hepatocellular carcinoma (HCC), the most common cancer in Taiwan [46]. AFP concentration is a powerful indicator in the assessment of HCC prognosis because the serum AFP concentration is markedly increased in patients with HCC [47]. We have detected AFP of various concentrations by using the SC SiNB FET sensors [36]. The best sensitivity of this sensor, which was obtained by biasing at a maximum of transconductance, was around 1.02, and the detection limit was estimated to be 100 fg/mL. Figure 9 presents the real-time drain current shift of the SC SiNB FET biosensor device when sensing 10 ng/mL AFP under different buffer concentrations. The SiNB FET sensor without surface modification served as a negative control in this experiment, and the current shift remained almost the same under varying buffer concentrations (the black line). By contrast, for the biosensor with APTES and GA surface modification, the drain current was strongly dependent on the buffer concentration. At first, 10X PBS but without AFP was injected into the sensors to measure the basic current. As presented in the figure, the difference of the current shift increased with decreasing buffer concentration, even when the AFP concentration remained the same (blue line). This result indicates that the Debye screening length is related to the sensitivity of the SC SiNB FET sensor even at the same biomolecule target concentration.

## 4. Conclusions

An SC SiNB FET was successfully fabricated for solution pH sensing. To enhance the sensitivity of the sensor, a lateral oxidation technique was employed to reduce the width of the silicon nanobelt from 350 to 150 nm. The pH detection results demonstrated that the SiNB FET sensor exhibited a stepwise change and linearity for solutions from pH 6.4 to 7.4 and remained stable, returning to the same current level at the same pH value after sensing different buffer solutions. The sensitivity of the SiNB FET sensor was dependent on the Debye screening length of the buffer solution. When the Debye length decreased and the ion concentration of the buffer increased, the sensitivity of the SiNB FET sensor decreased. This finding was also demonstrated in the real-time detection of AFP antigen by using the SiNB FET sensor. The obtained results indicated that the SC SiNB FET served as a sensitive and reliable sensor platform for pH and biomolecule sensing.

## Figures and Tables

**Table 1:** Comparison of pH sensitivity using nanowire based FET sensors.

pH Sensitivity	Nanowire Materials	Ref.
178 mV/pH	Poly-Si nanowire	[41]
56.3 mV/pH (single gate) 143.7 mV/pH (double gate)	Si nanowire	[42]
55.8 mV/pH	Si nanowire	[43]
42 mV/pH	Si nanowire	[44]
48.34 mV/pH	CuO	[45]
42.2 nA/pH (for 0.1X buffer)	Si nanowire	Our work
